# High Resolution Human Eye Tracking During Continuous Visual Search

**DOI:** 10.3389/fnhum.2018.00374

**Published:** 2018-10-02

**Authors:** Jacob G. Martin, Charles E. Davis, Maximilian Riesenhuber, Simon J. Thorpe

**Affiliations:** ^1^Centre de Recherche Cerveau & Cognition, CNRS-Université Toulouse III, Toulouse, France; ^2^Department of Neuroscience, Georgetown University Medical Center, Research Building, Washington, DC, United States

**Keywords:** visual search task, eye movements, face perception, gaze control, saccadic eye movements, dataset, human neuroscience

## Abstract

While several studies have shown human subjects’ impressive ability to detect faces in individual images in paced settings (Crouzet et al., 2010), we here report the details of an eye movement dataset in which subjects rapidly and continuously targeted single faces embedded in different scenes at rates approaching six face targets each second (including blinks and eye movement times). In this paper, we describe details of a large publicly available eye movement dataset of this new psychophysical paradigm (Martin et al., 2018). The paradigm produced high-resolution eye-tracking data from an experiment on continuous upright and inverted 3° sized face detection in both background and no-background conditions. The new “Zapping” paradigm allowed large amounts of trials to be completed in a short amount of time. For example, our three studies encompassed a total of 288,000 trials done in 72 separate experiments, and yet only took approximately 40 hours of recording for the three experimental cohorts. Each subject did 4000 trials split into eight blocks of 500 consecutive trials in one of the four different experimental conditions: {upright, inverted} × {scene, no scene}. For each condition, there are several covariates of interest, including: temporal eye positions sampled at 1250 hz, saccades, saccade reaction times, microsaccades, pupil dynamics, target luminances, and global contrasts.

## Background and Summary

We present here a large dataset of visual search in humans, which investigated the number of faces which could be targeted with the eyes each second in embedded and changing scenes continuously and without any experimental pauses between trials. Using a gaze-contingent paradigm with a high definition eye-tracker, we presented detection and targeting tasks to the subject as rapidly as they could solve them. As soon as the subject’s eye reached the target face, the next detection task was presented based on the previous target face’s location. The experiment sought to determine whether the visual system works as fast continuously as has been observed in paced experiments (Crouzet et al., 2010), and also whether the continuous detection rate is faster for particular types of targets (i.e., upright faces) versus that found for other types of visual targets (Wolfe, 2010; Wu and Kowler, 2013).

The methods and text of this paper are modified and expanded versions of the descriptions and text of our related work (Martin et al., 2018), wherein we used the dataset to show that subjects could continuously perform ultra-rapid face detection and localization with saccades at surprisingly fast sustained rates (targeting rates of up to 5.4 face targets/second when faces were hidden in a background, and up to six face targets/second when there was no background). This previous work investigated continuous saccadic search in the context of either pasting and/or blending the faces into different backgrounds, or pasting them on a simple gray background. Second, we used inverted faces as controls to test for advantages in search for upright faces (Kanwisher et al., 1998; Haxby et al., 1999; Yovel and Kanwisher, 2005; Kanwisher and Yovel, 2006). The results from the previous paper verified hypotheses that humans can continuously saccade toward hidden objects like faces at high rates, that upright faces are detected faster and more accurately than inverted faces in both background and no-background conditions, and that a saliency only model of saccade execution does not appear to completely explain continuous human saccadic behavior (Martin et al., 2018).

The data described in the current paper encompass human eye movements from a series of behavioral eye-tracking studies that were intended to extend our understanding of the efficiency of the neural mechanisms underlying continuous and rapid object recognition. The data contains a very rich set of covariates from 288,000 trials encompassing three experimental manipulations (full screen search, limited eccentricity search, blended limited eccentricity search). We have included pupil sizes, microsaccades, saccades, stimulus properties (local contrast, luminance), and 1250 hz eye position data according to each condition for each trial. As it would take an enormous amount of time to investigate all of these covariates and their relationships with the continuous dynamics of visual search, we have decided to release the dataset to the research community. We hope that the dataset will be of interest to a wide range of researchers and experimental disciplines who are interested in visual search and saccadic execution, and will provide a solid public dataset for future scientific exploration into the dynamics of continuous visual search.

## Methods

**FIGURE 1:**
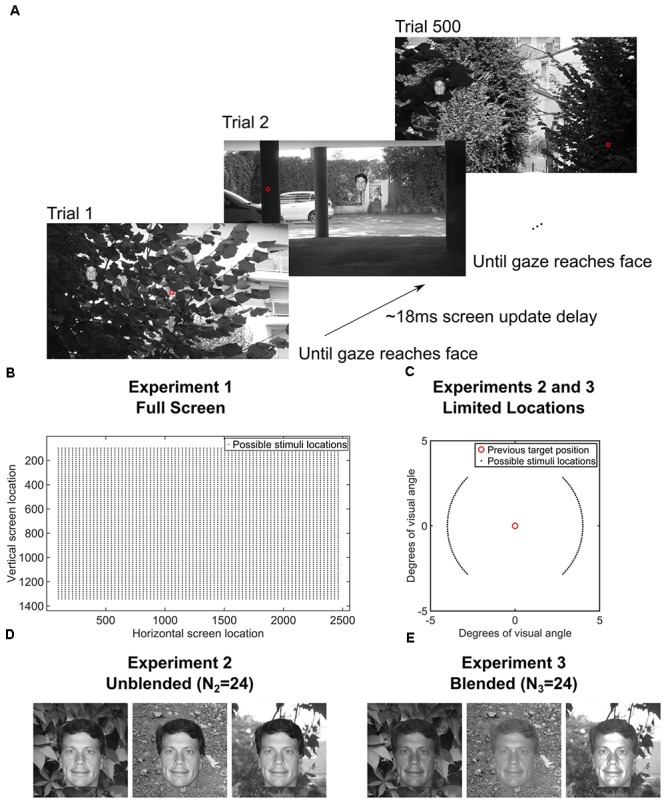
**(A)** Paradigm for the entire screen (Experiment 1, Data Citation 1). One block of 500 trials of the gaze-contingent continuous saccade detection paradigm with upright faces pasted on a background. The red circle is the gaze location and was not shown during the experiment. The subject had to localize the face with their gaze for the next trial to be presented. The face shown is just for illustration; faces and backgrounds within a block were different and were never repeated within each block of 500 trials *(*Martin et al., 2018*)*. **(B)** Locations of the 3° faces on each trial in Experiment 1 were independent from the previous trial and appeared at any location on the screen. **(C)** In Experiments 2 and 3, the locations of the 3° faces were within 4° eccentricity of the location of the face in the previous trial and appeared at polar angles between 0°–45°, 135°–235°, and 315°–360° of the previous face (Experiments 2 and 3, Data Citation 2, Data Citation 3). **(D)** Examples of how unblended faces would appear on different backgrounds in Experiment 2 (*N*_2_ = 24 subjects, Data Citation 2) (Martin et al., 2018). **(E)** Examples of how blended faces would appear on different backgrounds in Experiment 3 (*N*_3_ = 24 subjects, Data Citation 3) (Martin et al., 2018). **Figure 1C** is a modified reproduction from the source’s **Figure 3A** (Martin et al., 2018). **Figures 1D,E** are unmodified reproductions from the source’s **Figures 3B,C** (Martin et al., 2018). Some text from the source was duplicated and/or modified (Martin et al., 2018).

We ran three separate experiments designed to explore the speed of continuous saccadic face detection (*N*_1_ = 24 subjects, *N*_2_ = 24 subjects, *N*_3_ = 24 subjects). In all experiments, subjects continuously localized 500 different inverted or upright faces in counterbalanced blocks (**Figure 1A**). There were eight blocks, and each block had 500 different upright or inverted faces which were either *directly pasted* into one of 500 different cluttered background scenes or were pasted only on a gray screen as the background. No background scenes contained faces before we pasted the face stimulus into it. Moreover, faces and background scenes were never repeated during each 500 trial block. Whereas previous works have found that interleaving a blank screen, or “gap,” between the fixation cross and the onset of the visual target provokes faster saccades (Fischer and Weber, 1993), the paradigms in the current study did not have a fixation cross, did not have a 200ms “gap” before the next trial (as in Crouzet et al., 2010), did not have pauses between trials, there was no extensive training, and the subjects had no exact foreknowledge of the position of the target in each trial. As soon as the subject’s gaze arrived within a small square surrounding the face on the screen, the next trial was presented in a median of 18 ms (**Figure 1A**). More specifically, to move on to the next trial during the experiment, the stimulus computer had to continuously communicate with the eye tracker computer to detect whether the subject’s gaze was located within a square of size 3° × 3° centered on the face, and then update the screen with the next trial. During analysis after the experiment, we considered that the first saccade after stimulus onset was correct if and only if it landed within the 3° × 3° square centered on the face.

In Experiment 1 (Data Citation 1), each trial contained a single 3° face pasted at any possible location on the screen, covering eccentricities from 4° to 20° from the previous fixation. Because the gaze potentially had to travel across the entire screen in Experiment 1, we did another study (Experiment 2, Data Citation 2), which was designed to increase the number of faces that could be processed each second by presenting each subsequent face at a fixed eccentricity of 4° from the previous face (see **Figure 1B**). Also, the polar angles in Experiment 2 were set such that subsequent targets appeared at polar angles of 0°–45°, 135°–235°, 315°–360° from the previous target (**Figure 1B**, inset). To control for potential pop-out effects, we conducted a third experiment (Experiment 3, Data Citation 3) in which each face was further hidden in the scene by matching each face’s grayscale histogram with that of the local histogram in the background image in which it was pasted (**Figures 1C,D**). As in Experiment 2, each face was pasted at an eccentricity of 4° from the previous face.

### Stimuli

The face stimuli were cropped from a set of 2316 images of segmented faces from the Humanae project (with written permission from the artist and on the web at: http://humanae.tumblr.com/) (Dass, 2016). Unfortunately, we were not able to redistribute these images in the dataset. However, individual face images can be accessed on the photographer’s Tumblr website (currently located at http://humanae.tumblr.com/). Image filenames from the face images on the photographer’s website correspond to the filenames stored in the data.stimulusImageFile array (see **Table 2**). Faces were converted to grayscale before the experiment. Faces were resized to 3° of visual angle in height. We have included the pixel locations for seven facial landmarks for each trial (head top center, forehead top center, left eye, right eye, nose apex, mouth middle, and bottom chin middle).

Background scene image stimuli for all experiments were selected from a large database of images containing 861 images, some of which have been used in previous psychophysical studies (Thorpe et al., 1996). Luminance and global contrast (with respect to the pasted face) of the background scene images are available in the dataset. Image backgrounds were resized using the imresize function of MATLAB to cover the entire screen resolution of 2560 × 1440 pixels. The original image backgrounds varied in original size but almost always had an initial size greater than 1920 × 1080 pixels before resizing.

### Presentation Hardware

Stimuli were presented on an ASUS ROG Swift PG278Q GSYNC monitor with 1 ms response time and 120 Hz refresh rate at a screen resolution of 2560 × 1440 pixels. The size of screen in cm was 61.5 cm width × 35.5 cm height. Subjects’ eyes were at an approximate distance of 85.0 cm to the screen (as measured with a laser distance measure device with distance measurement precision of ±2 mm). The display therefore subtended approximately 31° horizontal and 22° vertical of visual angle. There was no gamma correction done during image presentation. The default parameters for the monitor were used. We have included data on the luminance of each background image. The display was controlled by a custom-built workstation running Gentoo Linux with a 64 bit kernel that was tuned for real-time processing. The paradigm was programmed in MATLAB R2008a (The Mathworks, MA) using Psychtoolbox version 3 (Brainard, 1997; Pelli, 1997). Target onset presentation times were recorded using a photodiode that was linked with the high-speed analog input of the eye-tracker recording through the Cedrus StimTracker system.

### Eye-Tracking Hardware

Eye movements were recorded using the SMI iViewX High Speed system with a 1250 hz sampling rate on the subject’s master eye. Before the first session, we determined each subject’s dominant eye and subsequently recorded and calibrated that eye. The eye-tracker recorded the dominant eye at a rate of 1250 hz and sent gaze position samples with a processing delay of approximately 2 ms to the presentation hardware. We used the “microsacc plugin” for saccade detection with a smoothing level of 2 [“the raw data is smoothed over a five-sample window to suppress noise”(Engbert and Mergenthaler, 2006)], a velocity factor of λ = 5 to determine the velocity threshold for saccade detection [“thresholds were relative to the noise level, calculated as λ = 5 multiples of a median-based SD estimator” (Engbert and Mergenthaler, 2006)], and a minimum saccade duration of 10 samples (corresponding to 8 ms) (Engbert and Mergenthaler, 2006). For each trial, the saccade data for every saccade on that trial is available in the “saccades” field. Likewise, the saccades for each trial as determined by the default SMI saccade detection algorithm are available in the “smisaccades” structure field. Sample data for eye movements include the following: raw position data, saccade reaction times, saccade accuracies, pupil sizes, microsaccades, eccentricities, polar angles, and more (see **Table 2**). Pupil diameter values are based on the number of pixels, not the number of millimeters. A conversion to millimeters was calculated as instructed by the SMI iViewX manual obtaining a conversion factor of “8.11.” However, the resolution of the pupil data is only as good as the SMI iViewX Hi-speed 1250 hz system and the conversion factor. Unfortunately, while pupil sizes were calibrated before the experiments as described in the iViewX manual, we could not find any reported accuracy levels of pupil size for this SMI system, and do not have any way to verify them in a continuous setting. However, a consistent calibration of the circle around the pupil was done before each block and lighting conditions were the same for each subject.

### Experimental Design

A total of 44 subjects with normal or corrected-to-normal vision participated in a total of 72 separate sessions divided into three experiments: Experiment 1 [*N*_1_ = 24, two left-handed, 14 females, ages 21–39 (Data Descriptor 1)], Experiment 2 [*N*_2_ = 24, two left-handed, 10 females, ages 22–53 (Data Descriptor 2)], and Experiment 3 [*N*_3_ = 24, two left-handed, 13 females, ages 21–40 (Data Descriptor 3)]. Some subjects participated in more than one of the three experiments: 6 took part in all three experiments, 9 in only Experiments 1 and 2, 6 in only Experiments 1 and 3, 1 in only Experiments 2 and 3, 3 in only Experiment 1, 8 in only Experiment 2, and 11 in only Experiment 3. Subjects within each experiment were unique, but some subjects did multiple experiments. Each experiment consisted of 4 different conditions [(1) No Background, Upright; (2) No Background, Inverted; (3) Background, Upright; (4) Background, Inverted)]that were separated into separate blocks. Each block contained 500 trials all of a single condition. The orders of the blocks were counterbalanced across subjects so that each of the subjects did one of the possible 24 possible block orderings of the four conditions. This same order of four blocks was then repeated in another section of four blocks, so that subjects did a total of eight blocks. After each block of 500 trials, there was a small pause of about 2 min while the eye tracker was recalibrated to ensure that the calibration remained accurate throughout the experiment.

Within each block, participants performed a continuous detection task on the 3° face stimuli (see **Figure 1**). Each subsequent trial started immediately after the subject’s eye landed within the previous face (with a mean screen-update error of 18.03 ms after the subject found the previous face – see **Figure 1**). Faces were randomly pasted either based on the position of the face in the previous trial (Experiments 2 and 3) or completely randomly within the 2560 × 1440-pixel scene (Experiment 1). All faces and background images within any given block were unique. The faces and background images were created randomly before the block and were counterbalanced across subjects such that each face and background image combination appeared equally in each condition across subjects. Participants were told to find the faces with their eyes as fast as they possibly could.

### Code Availability

**FIGURE 2:**
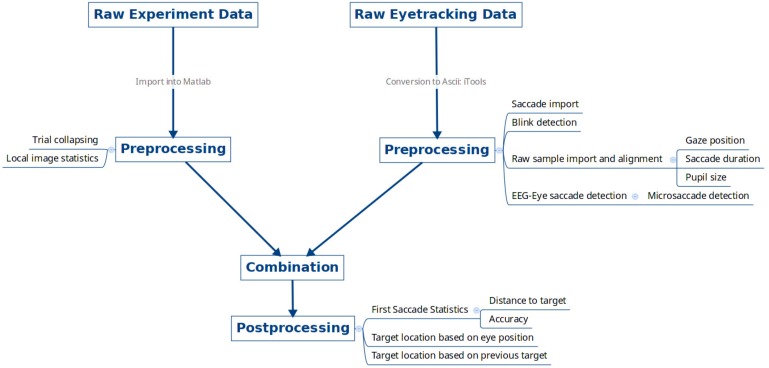
Data pre-processing overview. Raw experimental data files for each subject from the psychophysics computer and raw eye tracking data were exported, pre-processed, and combined together.

The data were imported, collated, and pre-processed using the flowchart shown in **Figure 2**. The following list contains the versions and parameters used from each pre-processing utility.

• SMI iViewX Hi-Speed 1250 hz

   ◦ Saccade detection:

      • Event Detector (v3.0.20)

            • Minimum Duration: Auto
            • Minimum Fixation Duration: 5 ms
            • Peak Velocity Threshold: 20°/s
            • Peak Velocity Start: 20% of saccade length
            • Peak Velocity End: 80% of saccade length

• Psychtoolbox (v3.0.12)
• Matlab (R2017b)
• EYE-EEG: (v0.41), microsacc_plugin (v2.1) (Engbert and Mergenthaler, 2006; Dimigen et al., 2011)

   ◦ Parameters

            • Velocity Factor: 5
            • Minimum Saccade Duration: 10 samples
            • Smoothing Level: 2
            • Global Velocity Threshold: Yes
            • Microsaccade size limit: 1°

• Saccade statistics

   ◦ Accuracy defined as whether the first saccade landed within a square of size 150px centered on the center of the face image.

• Image statistics

   ◦ Target luminance:

            • The mean of the gray levels of the pasted pixels of the face.

   ◦ Global contrast:

            • The mean of the gray levels of the pixels of the background image minus the mean of the gray levels of the pasted pixels of the face.

The selectIndices() utility script is available for download from the Figshare website.

### Data Records

All data and code corresponding to the data citations are stored on the Figshare data publishing site and are accessible from the Internet (Data Citation 1, Data Citation 2, Data Citation 3, Data Citation 4, see **Table 1**). The datasets are provided as three separate MATLAB (Mathworks^TM^) “.mat” data files for each of the three experiments. Each file contains the subject numbers for the subjects in the corresponding experiment (which are unique to a subject across experiments), so that one can investigate both within and between subject factors by simply collating on the subject numbers across experiments. The datasets use an indexed approach for ease of access. The fields of the dataset are described in **Table 2**. For example, to compute the accuracy of upright faces for subject with id 5, one can simply execute the MATLAB command:

**Table 1:** Experimental studies summary.

Source	Sample	Sample Number	Details	Protocol 1	Protocol 2	Protocol 3	Protocol 4
https://doi.org/10.6084/m9.figshare.5549362	Dataset 1: martin_zap _experiment1.mat	24 subjects, 96,000 trials	3° faces at 4^∘-^20° eccentricities, unblended	No Scene, upright face	No Scene, inverted face	Scene, upright face	Scene, inverted face
https://doi.org/10.6084/m9.figshare.5549362	Dataset 2: martin_zap _experiment2.mat	24 subjects, 96,000 trials	3° faces at 4° eccentricities, unblended	No scene, upright face	No scene, inverted face	Scene, upright face	Scene, inverted face
https://doi.org/10.6084/m9.figshare.5549362	Dataset 3: martin_zap _experiment3.mat	24 subjects, 96,000 trials	3° faces 4° eccentricities, blended	No scene, upright face	No scene, inverted face	Scene, upright face	Scene, inverted face
https://doi.org/10.6084/m9.figshare.5549362	Dataset 4:selectIndices.m	Code	Code for selecting subsets of data	NA	NA	NA	NA

**Table 2:** Data variable name descriptions.

Variable Name	Short description
trialnumber	Trial number in the experiment (1–4000)
sessionnumber	Session number in the experiment (1–8)
targeteccentricity	Eccentricity of the target relative to the previous target
targeteccentricityoriginal	Eccentricity of the target relative to the center of the screen
targetpolarangle	Polar angle of the target relative to the previous target
targetpolarangleoriginal	Polar angle of the target relative to the center of the screen
sessionIndex	Block number in the experiment (1 to 8)
trialIndex	Trial number in the block (1 to 500)
useBackground	Whether or not there was a background scene (1 = scene, 0 = gray background)
invertedStimulus	Whether or not the face stimulus was inverted (1 = inverted, 0 = upright)
pastedXYLocation	Location of the center of the face stimulus on the screen
stimulusEccentricity	Eccentricity of the target relative to the previous target
stimulusPolarAngle	Polar angle of the target relative to the previous target
subjectHasRightAnswer	Whether or not the first saccade after stimulus onset landed within a 3x3 square surrounding the face stimulus.
triggeronsets	Timestamp at which the stimulus was shown on the screen. All eye movement sample data for each trial is aligned on this timestamp (with a -200ms of presimulus data). These trigger onsets can be simply collated with the eye sample data using the first column of the eyedata.sampledata field. Note that trigger onsets times and sample times are particular to each subject.
zaptargeteccentricity	Eccentricity of the target relative to the current gaze location of the eye
zaptargetpolarangle	Polar angle of the target relative to the current gaze location of the eye
subjectnumber	The unique identifier for each subject. Subject identifiers are unique across the entire 72 subjects.
numMicroSaccades	Number of microsaccades within -200 to 800 ms of the trial.
numMicroSaccadesBeforeStimulus	Number of microsaccades within -200 to -1 ms of the trial.
numMicroSaccadesAfterStimulus	Number of microsaccades within 0 to 800 ms of the trial.
distanceToEdgeX	Distance in pixels of the center of the target to the closest horizontal edge of the screen
distanceToEdgeY	Distance in pixels of the center of the target to the closest vertical edge of the screen
eyedata.saccades	Contains data for each saccade in the trial as detected by EEG-EYE (starttime: ms after stimulus onset; startx and starty: x and y location of the start of the saccades; endx and endy: x and y location of the endpoint of the saccades).
eyedata.saccadeReactionTimes	Reaction time of the saccade after stimulus onset (in ms) as computed by the microsacc_plugin.
eyedata.saccadeSides	Whether the saccade was leftward or rightward (0 = left, 1 = right, NaN = no saccades in trial)
eyedata.saccadePolarAngles	The polar angle of the saccade
eyedata.sampledata	Raw sample data of eye positions from -200 to 800 ms at 1250 hz. Matrix is 3D (96,000 trials, 9 data types, 1250 samples). The data type indices are (1 = timepoint in samples, 2 = raw X, 3 = raw Y, 4 = pupil diameter X, 5 = pupil diameter Y, 6 = corneal reflex x, 7 = corneal reflex y, 8 point of regard X (gaze position in screen coordinates), 9 = point of regard Y)
eyedata.saccadeDistanceToTarget	Distance of the saccade to the target in pixels
eyedata.startingEyePositionCol	Eye position in columns on the screen at stimulus onset
eyedata.startingEyePositionRow	Eye position in rows on the screen at stimulus onset
eyedata.saccadesUntilTarget	Number of saccades needed to find the target
eyedata.timeToFindTarget	Amount of time needed to find the target (in ms)
eyedata.timeToLeaveFixation	Amount of time to leave the previous fixation zone (in ms).
eyedata.timeIndexFoundTarget	The eyedata.sampledata(:,1,:) time index in which the target was reached.
eyedata.smiSaccadeReactionTimes	Saccade reaction time as computed by the SMI iTools algorithm
eyedata.microsaccades	Microsaccade data: 1: onset of microsaccade (in ms after stimulus onset), 2: end of microsaccade (in ms after stimulus onset), 3: peak velocity of microsaccade, 4: horizontal component (dx), 5: vertical component (dy), 6: horizontal amplitude (dX), 7: vertical amplitude (dY), 8: euclidean distance of travel (pixels)
eyedata.microsaccadeblips	Matrix of ones and zeros where one means a microsaccade was detected and zero means no microsaccade was detected in each trial (rows) at each timepoint (columns). (96000 trials, 1250 samples)
eyedata.saccadesblips	Matrix of ones and zeros where one means a saccade was detected and zero means no saccade was detected in each trial (rows) at each timepoint (columns). (96,000 trials, 1250 samples)
eyedata.velocityratesx	X-component of the eye velocity rate at each timepoint of the trial
eyedata.velocityratesy	Y-component of the eye velocity rate at each timepoint of the trial
eyedata.saccadePeakVelocities	Peak velocity of the first saccade after stimulus onset
eyedata.saccadeAmplitudeX	Amplitude (in pixels) of the x-component of the first saccade after stimulus onset
eyedata.saccadeAmplitudeY	Amplitude (in pixels) of the y-component of the first saccade after stimulus onset
eyedata.saccadeDuration	Duration of the saccade (in ms)
eyedata.smisaccades	Data for each of the saccades in each trial after stimulus as computed by the SMI iTools algorithm.
eyedata.numSaccadesBeforeStimulus	Number of saccades in the 200ms before stimulus onset
eyedata.numSaccadesAfterStimulus	Number of saccades in the 800ms after stimulus onset
eyedata.saccadeAmplitudePixels	The amplitude (in pixels) of the first saccade after stimulus onset
eyedata.alleyevelocities	The velocity components during the -200ms to 800ms period of the trial (x-component = first column and y-component = second column)
eyedata.blinkPrestimulusTime	Time (in ms) of any blinks which occurred before the stimulus onset during the period -200 to 0 ms. NaN means there were no blinks.
imagestatistics.targetLuminance	The luminance of the target (mean of the gray level values).
imagestatistics.globalContrast	The contrast of the target
imagestatistics.stimulusImageFile	The filename of the face used as a stimulus.
imagestatistics.stimulusBackgroundFile	The filename of the background scene. Note that if useBackground = 0, there was no background scene actually painted, but in histogram-blended conditions (Experiment 3, Data Citation 3), this scene image file and location were used to blend the histogram.
imagestatistics.highIntensity	Whether or not the luminance was bigger than the mean luminance of all trials in the experiment (1 = greater than average luminance, 0 = less than average luminance)
imagestatistics.globalContrast	Global contrast of the background image with respect to the pasted face. Concretely, we subtracted the mean luminance of the face from the mean luminance of the entire background image.
imagestatistics.backgroundLuminance	Global luminance of the background image.
imagestatistics.facelocations	Structure containing the row and column locations of seven facial landmarks for each particular trial.

mean(data.subjectHasRightAnswer(data.subjectnumber == 5 & data.invertedStimulus == 0));

The helper function selectIndices() (in Data Citation 4) facilitates the selection of all the data with indices of a given type. For example:

inverteddata = selectIndices(data,data.invertedStimulus == 1);

The structure of data is indexed by trial. For each experiment, there is a single data structure for each of the 24 subjects recorded. To access information about a particular trial, one chooses the corresponding field of the main experimental data structure like so, e.g., trial 55:

data.targeteccentricity(55);

Generally, saccade information is stored in the “eyedata” field. For example, the onset time of the third saccade of the fifth trial in the entire experimental cohort is accessible like so:

data.eyedata.saccades{5}.starttime(3)

Raw sample data for eye movements are contained in the “data.eyedata.sampledata” field. This field contains the raw sample data (timepoint, raw x, raw y, diameter x, diameter y, corneal reflex x, corneal reflex y, point of regard x, point of regard y) for the 200 ms (250 samples at 1250 hz) before the stimulus onset and the 800ms after and including stimulus onset (see **Table 2**).

While access to the data is trial-oriented, one can ascertain which other images were presented during a particular section of eye data by using the following procedure (for example, trial 2690 of subject 9):

datasubject = selectIndices(data,data.subjectnumber == 9); % trigger onsetsare particular to subjects
  Trial number = 2690; %
  trialIndicesDuringOneSecondPeriodAroundTrial = find (datasubject. eyedata.triggeronsets> =min(squeeze(datasubject.eyedata.sampledata (trialnumber,1,:))) & datasubject.eyedata.triggeronsets <= max(squeeze (datasubject.eyedata.sampledata(trialnumber,1:,:)))));
  imageFilesDuringOneSecondPeriodAroundTrial = datasubject.stimulus ImageFile{trialIndicesDuringOneSecondPeriodAroundTrial}

Using this same method, one can access any other desired data of interest. For example, to obtain the target luminances around a particular trial:

targetLuminancesDuringOneSecondPeriodAroundTrial=datasubject. imageproperties.targetLuminance(trialIndicesDuringOneSecondPeriod AroundTrial);

We exported the trial onset time data for each trial into a field called “eyedata.triggeronsets.” These trial onset times can be simply collated with the eye data using the eyedata.sampledata field.

### Technical Validation

We first verified that the data were similar to that in previous experiments on human eye movements (saccade peak velocity vs. saccade duration, main sequence, saccade reaction time, microsaccade rates, etc.). Stimulus timing was recorded and validated by a photodiode connected to the screen and put into the SMI system via a high-speed analog card link. Stimulus timing was also duplicated with Ethernet messages. SMI iViewX calibration was performed after each of the eight 500 trial blocks. SMI accuracies are reported in the system’s manual and also in scientific papers (Mantiuk, 2016). We used the standard automatic SMI 13-point calibration/validation procedure, which, according to the SMI manual, typically results in gaze position accuracies of 0.25°–0.5° of visual angle. This procedure accepted each point automatically based on the average of 400 ms of data for each point. In addition, there was audio feedback (a beep) when each new point was displaced. As described in the SMI iViewX manual (p. 148 – Calibration Setup), we randomized the order of the display of the points, set the system to “Wait for Valid Data” (which enforced the detection of a valid fixation before proceeding), and set the SMI “Check Level” for each point to “Medium” (which determined “how strict the system was in accepting calibration points and overall geometry”) (Mantiuk, 2016).

For the analysis of experimental error and variation, the timing of processing and communication between eye-tracking system and experimental computer configuration was done via an oscilloscope and network latency tests. We recorded eye movements using the SMI iViewX High Speed system with a 1250 Hz sampling rate on the subject’s dominant eye. Before the first session, we determined each subject’s dominant eye and subsequently recorded and calibrated that eye. The eye-tracker recorded the dominant eye at a rate of 1250 Hz and sent gaze position samples at a delay of approximately 5 ms to the presentation hardware. We compared the time of the entrance of the eye within the target face area and the subsequent photodiode onset for the next trial, we were able to identify that the median screen update time was 18.03 ms. These values did not differ by more than 1 ms when examined by condition (e.g., for Experiment 1: 17.67 s, 17.95 ms, 18.09 ms, and 18.42 ms).

### Exclusion Criteria

One out of 74 initial subjects quit the experiment pre-maturely due to discomfort, and this subject’s data were removed from the dataset. Another subject that had problems with eye-tracking calibration (in which case the experiment was prematurely terminated) was also excluded. To complete the experimental cohort, two additional subjects performed the full experiment to complete the planned 24 subjects per experiment. These exclusion criteria were established before the study was conducted.

### Randomization and Blinding

Face-to-condition assignments and target locations were performed randomly for each subject and block. A block design was used in order to counterbalance 24 condition block orders across 24 subjects. The experimenter who set up and administered the experiment was blind to the condition order.

### Animal and Human Studies

The Committee for the Evaluation of Ethics of INSERM (CEEI Institutional Review Board) approved the experimental procedures and protocol, and written informed consent was obtained from all participants prior to each experiment. All experiments were performed in accordance with relevant guidelines and regulations.

## Data Citations

(1) Martin, J. G., Davis, C. E., Riesenhuber, M., and Thorpe, S. J. (2018). *Figshare.* Available at: https://figshare.com/s/26f064e4a30322fbcc03_D2
2) Martin, J. G., Davis, C. E., Riesenhuber, M., and Thorpe, S. J. (2018). *Figshare.* Available at: https://figshare.com/s/26f064e4a30322fbcc03_D6
(3) Martin, J. G., Davis, C. E., Riesenhuber, M., and Thorpe, S. J. (2018). *Figshare.* Available at: https://figshare.com/s/26f064e4a30322fbcc03_D7
(4) Martin, J. G., Davis, C. E., Riesenhuber, M., and Thorpe, S. J. (2018). *Figshare.* Available at: https://figshare.com/s/42f14f2d92d994fd8fb4

## Author Contributions

JM and ST conceived the experimental protocol. JM, ST, and MR proofread the paper. JM wrote the main manuscript text, analyzed, processed, and prepared all figures and data. CD recruited and ran human participants, recorded face landmarks, and helped proofread the paper.

## Conflict of Interest Statement

The authors declare that the research was conducted in the absence of any commercial or financial relationships that could be construed as a potential conflict of interest. This article uses material which was licensed under a Creative Commons Attribution 4.0 International License, which permits use, sharing, adaptation, distribution and reproduction in any medium or format, as long as one gives appropriate credit to the original author(s) and the source, provides a link to the Creative Commons license, and indicates if changes were made.
